# Blood-feeding patterns of native mosquitoes and insights into their potential role as pathogen vectors in the Thames estuary region of the United Kingdom

**DOI:** 10.1186/s13071-017-2098-4

**Published:** 2017-03-27

**Authors:** V. A. Brugman, L. M. Hernández-Triana, M. E. England, J. M. Medlock, P. P. C. Mertens, J. G. Logan, A. J. Wilson, A. R. Fooks, N. Johnson, S. Carpenter

**Affiliations:** 1 The Pirbright Institute, Ash Road, Woking, Surrey, UK; 2 London School of Hygiene and Tropical Medicine, Keppel Street, London, UK; 3 Animal and Plant Health Agency, New Haw, Addlestone, Surrey, UK; 4 Public Health England, Porton Down, Salisbury, UK; 5 Health Protection Research Unit in Emerging Infections & Zoonoses, Porton Down, Salisbury, UK; 6 The University of Nottingham, Sutton Bonington, Leicestershire, UK; 7 Department of Clinical Infection, Microbiology and Immunology, University of Liverpool, Liverpool, UK; 8 Faculty of Health and Medical Sciences, University of Surrey, Guildford, Surrey, UK

**Keywords:** Mosquito, Blood-meal, Feeding patterns, Pathogen, Sella scale, Migratory birds

## Abstract

**Background:**

The range of vertebrate hosts on which species of mosquito blood-feed is an important parameter for identifying potential vectors and in assessing the risk of incursion and establishment of vector-borne pathogens. In the United Kingdom, studies of mosquito host range have collected relatively few specimens and used techniques that could only broadly identify host species. This study conducted intensive collection and analysis of mosquitoes from a grazing marsh environment in southeast England. This site provides extensive wetland habitat for resident and migratory birds and has abundant human nuisance biting mosquitoes. The aim was to identify the blood-feeding patterns of mosquito species present at the site which could contribute to the transmission of pathogens.

**Methods:**

Twice-weekly collections of mosquitoes were made from Elmley Nature Reserve, Kent, between June and October 2014. Mosquitoes were collected using resting boxes, by aspiration from man-made structures and using a Mosquito Magnet Pro baited with 1-octen-3-ol. Blood-fed specimens were classified according to the degree of blood meal digestion using the Sella scale and vertebrate origin determined using sequencing of a fragment of the mitochondrial cytochrome C oxidase subunit I gene. Mosquitoes that were morphologically cryptic were identified to species level using multiplex PCR and sequencing methods.

**Results:**

A total of 20,666 mosquitoes of 11 species were collected, and 2,159 (10.4%) were blood-fed (Sella scale II-VI); of these 1,341 blood-fed specimens were selected for blood meal analysis. Vertebrate origin was successfully identified in 964 specimens (72%). Collections of blood-fed individuals were dominated by *Anopheles maculipennis* complex (73.5%), *Culiseta annulata* (21.2%) and *Culex pipiens* form *pipiens* (10.4%). Nineteen vertebrate hosts comprising five mammals and 14 birds were identified as hosts for mosquitoes, including two migratory bird species. Feeding on birds by *Culex modestus* and *Anopheles atroparvus* populations in England was demonstrated.

**Conclusions:**

This study expands the vertebrate host range of mosquitoes in the Thames estuary region of the UK. Feeding on both resident and migratory bird species by potential arbovirus vectors including *Cx. pipiens* f. *pipiens* and *Cx. modestus* indicates the potential for enzootic transmission of an introduced arbovirus between migratory and local bird species by native mosquito species.

**Electronic supplementary material:**

The online version of this article (doi:10.1186/s13071-017-2098-4) contains supplementary material, which is available to authorized users.

## Background

Identifying the range of vertebrate hosts on which mosquitoes blood-feed is an important component of understanding vector-host-pathogen interaction dynamics and in determining the role of different mosquito species in inflicting biting nuisance on humans and animals [1]. The United Kingdom (UK) is considered at risk from exotic mosquito-borne pathogens of medical and veterinary importance. Several native mosquito species have been identified as proven or potential pathogen vectors [2–4], and on-going environmental changes may facilitate the establishment of exotic pathogens and vector species [5]. The detection of the eggs of *Aedes albopictus* Skuse in gravid traps in Folkstone, Kent in September 2016 exemplifies this threat [6].

Current information on the blood-feeding behaviour of UK mosquitoes is based on studies [7–17] that have collectively investigated the blood meals of 21 mosquito species and identified feeding on at least ten vertebrate hosts. The majority of these studies, however, were conducted prior to the development of molecular methods to delineate mosquito sibling species and, importantly, do not provide information on the utilisation of wild resident or migratory birds as blood-feeding hosts. Only one resident bird, the pigeon (*Columba* sp.), has previously been identified as a host [12]. Therefore, a need exists for further investigation into the avian hosts of mosquitoes in the UK in line with recent studies conducted in mainland Europe (e.g. [17–21]).

Identifying species of wild bird that are fed upon by mosquitoes is of particular importance given the role of birds as reservoir hosts of several important arboviruses in continental Europe. These arboviruses include West Nile, Usutu and Sindbis viruses (WNV, USUTV, SINDV) [22–24], antibodies to which have previously been reported from wild and sentinel birds in the UK [25, 26], although these results have not been supported by subsequent surveys [27–30]. The movement of infected migratory birds from endemic areas is considered to play a role in the introduction and spread of arboviruses such as WNV in mainland Europe (reviewed in [31]) and is considered a potential entry mechanism into the UK [29, 32].

The digestion of a blood meal within a mosquito rapidly reduces the probability of successful amplification of host marker DNA sequences. The Sella scale provides a simple and standardised visual method for determining the stage of blood meal digestion within a mosquito [33] and is useful to assess the period over which a given molecular method will be effective. For example, successful identification of blood meal host in field specimens collected in Spain was shown to decrease from 80–90% at Sella stage II to 5–25% at stage VI [34]. Relatively few studies use this simple classification system, however, limiting the conclusions that can be drawn regarding the effectiveness of a given molecular method over time. For instance, although a previous UK study successfully identified blood meal host in 46% of specimens [10], no information on the degree of blood meal digestion in each specimen was provided.

This study was conducted at Elmley Nature Reserve in Kent, a coastal grazing marsh environment in the southeast of England. Low-lying wetland areas of the Thames estuary region such as Elmley support resident and migratory bird populations and have a long association with high-density human-biting mosquito populations [35–37], as well as historical *Plasmodium vivax* transmission [38, 39]. Taken together, this region of the UK could provide favourable conditions for mosquito-borne pathogen establishment. The aim of this study was to identify the vertebrate blood meal hosts of mosquitoes at Elmley, in particular, to identify any avian species being fed upon by potential arbovirus vector species. To achieve this aim, mosquitoes were intensively collected using a combination of resting boxes and direct collection methods. Meteorological data were also collected from the site to assess the impact of weather variables on the collection of blood-fed specimens.

## Methods

### Mosquito collections

**Fig. 1:**
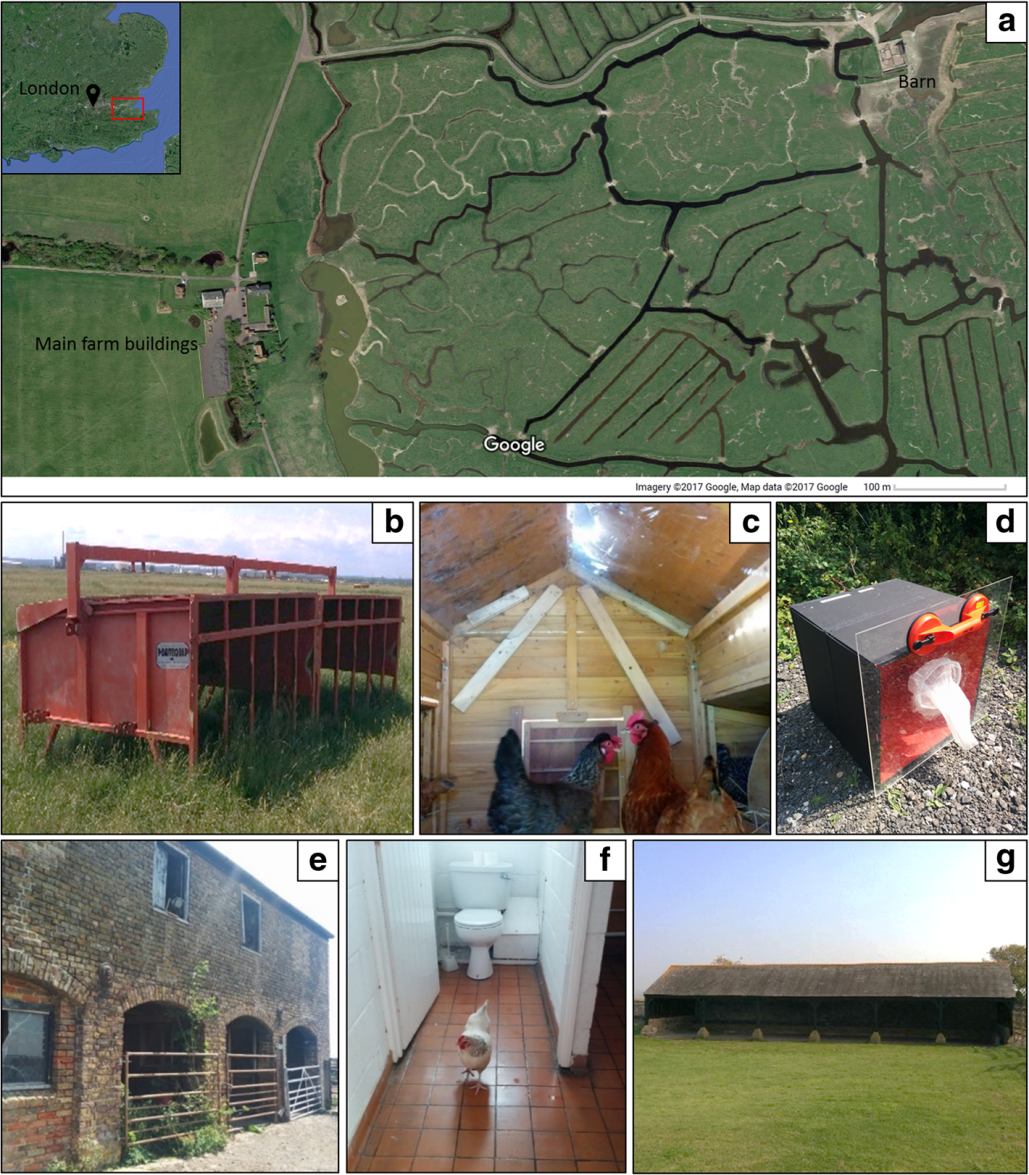
Site map and collection sites for resting mosquitoes at Elmley. (**a**) map of Elmley with insert showing relative location of the site in the Thames estuary, (**b**) red cattle feeder, (**c**) chicken coop, (**d**) the resting box with Perspex collection sheet, (**g**) disused cattle feeding shelter, (**f**) public toilets accessible to animals, (**e**) cattle barn

All mosquito collections were conducted at Elmley National Nature Reserve, Isle of Sheppey, UK (map reference point: 51.377445°N, 0.78406°E, Fig. 1). Mosquitoes were collected over two consecutive days per week for 36 weeks from June to October 2014. On each day, mosquitoes were aspirated from 20 resting boxes, distributed across four locations (denoted A, B, C and D) within an overall area of no more than 1 km^2^. In addition, mosquitoes were also collected from human-made structures (public toilets, two chicken coops, a cattle barn, a red cattle feeder and a disused feeding shelter: Fig. 1) using a manual aspirator (Model 612, John W Hock, Gainsville, Florida, USA) and a Prokopak backpack aspirator (John W Hock).

Resting boxes were made of 5 mm plywood with exterior dimensions W: 500 mm × H: 500 mm × D: 500 mm (‘No nail’ plywood boxes, Davpack, Derby, UK) and painted, after an initial primer coat, matt black on the outside and matt red on the inside (all paint from B&Q, Guildford, UK). A 5 mm clear acrylic sheet with dimensions W: 600 mm × H: 600 mm (Display Pro, Norfolk, UK) with a 120 mm diameter hole cut in the centre and covered with netting removed from a BugDorm insect cage (BugDorm, Taichung, Taiwan) was placed over the open face of the box to prevent mosquito escape during aspiration (Fig. 1).

Sequential collections from resting boxes and the public toilets were made during three time periods each day: between 08:30 and 09:30 (‘morning’), between 11:30 and 12:30 (‘noon’), and between 14:30 and 15:30 (‘afternoon’) to assess daytime recruitment into the boxes. Aspiration from the other habitats took place once per day between the above collections. A Mosquito Magnet Pro (MMP) trap baited with 1-octen-3-ol (Midgetech, Stirling, UK) was placed at a single location situated at least 200 m from the other collection locations and run overnight between the two collection days to provide an estimate of background mosquito populations.

### Processing of mosquitoes

Collected mosquitoes were placed into a cooler containing dry ice and transported to the laboratory for storage at−20 °C until processing. Identification to species/species group was initially based on morphological features following the keys of Snow [40] and Cranston [41]. The degree of blood meal digestion in blood-fed specimens was also classified using the scale of Sella [33] from stage two (II) (recently fed and fully engorged) to six (VI) (blood meal almost completely digested).

### DNA extraction and blood meal identification

DNA extraction and blood meal identification were conducted with slight modification from a methodology described previously by Brugman et al. [10]. Briefly, the abdomens of blood-fed specimens were squashed into 200 μl of phosphate buffered saline, using a three-stage wash (5% decon, 100% ethanol and sterile water) between specimens to avoid cross-contamination. DNA was extracted from the resulting solution using the DNeasy Blood and Tissue Kit (Qiagen, Manchester, UK) following the manufacturer’s spin column-protocol. Blood meal origin was determined using a six-primer cocktail (VF1_t1 + VF1d_t1 + VF1i_t1/VR1_t1 + VR1d_t1 + VR1i_t1) which targets a 685 base pair (bp) sequence of the *cox*1 gene [42] (all primer sequences used in this study are detailed in Additional file 1: Table S1). A slightly higher primer concentration was used than previously [10], as this was found to produce more consistent results. The final reaction mix (50 μl) consisted of: 28.075 μl H_2_O, 5 μl GeneAmp 10× PCR buffer I (Applied Biosystems, Life Technologies Ltd, Paisley, UK), 1 μl dNTPs (at 0.2 mM/μl), 1.5 μl of each primer (at 10 pmol/μl), 0.25 μl Ampli*Taq* Gold DNA Polymerase (10 units/μl) (Applied Biosystems, Life Technologies Ltd, Paisley, UK), 0.675 μl 100% dimethylsulfoxide (DMSO), 1 μl tetramethylammonium chloride (TMAC) at 2.5 mM and 5 μl extracted DNA. The thermal profile consisted of an initial denaturation step at 94 °C for 10 min followed by 40 cycles of 94 °C for 30 s, 45 °C for 30 s, 72 °C for 1 min, followed by a final elongation step of 72 °C for 10 min. PCR products were separated on a 1.5% agarose gel and positive amplification products sequenced unidirectionally with M13 primers [42] at 1 pmol/μl using the ABI PRISM® BigDye® Terminator v3.1 Cycle Sequencing Kit (Applied Biosystems). Sequences were assigned to a particular species when the agreement was ≥ 98% to known species in GenBank.

### Molecular identification of mosquito species

#### *Culex pipiens* (*s.l*.)/*Culex torrentium*

Mosquitoes morphologically identified as *Culex pipiens* (*s.l*.)/*Culex torrentium* were identified to species level using two sequential, end-point, PCR assays. First, *Cx. pipiens* (*s.l*.) was separated from *Cx. torrentium* using a duplex PCR assay [43] utilising the ACEtorr and ACEpip forward primers and B1246s reverse primer which target the nuclear acetylcholinesterase-2 (*ace-2*) gene [44]. The final reaction mix (10 μl) consisted of: 3 μl of DNA template, 0.4 μl nuclease-free H_2_O, 5 μl Top*Taq* Mastermix (Qiagen), 0.2 μl MgCl_2_ (Qiagen), 1 μl CoralLoad concentrate (Qiagen), 0.1 μl of each forward primer and 0.2 μl of the reverse primer (each at 10 pmol/μl). The thermal profile consisted of an initial denaturation step at 94 °C for 3 min followed by 35 cycles of 94 °C for 30 s, 55 °C for 30 s, 72 °C for 1 min, followed by a final elongation step at 72 °C for 10 min. Products were separated on a 1.5% agarose gel with expected product sizes of 610 bp for *Cx. pipiens* (*s.l*.) and 416 bp for *Cx. torrentium*.

Secondly, a duplex PCR assay was used to detect the presence of the two ecoforms of *Cx. pipiens* (*s.l*.), *Cx. pipiens* f. *pipiens* and *Cx. pipiens* f. *molestus*, using the forward primer CQ11F and reverse primers molCQ11R and pipCQ11R which target the CQ11 microsatellite locus [45, 46]. The final reaction mix (10 μl) consisted of 3 μl of DNA template, 0.325 μl nuclease free water, 5 μl Top*Taq* Mastermix (Qiagen), 0.2 μl MgCl_2_ (Qiagen), 0.075 μl bovine serum albumin (New England Biolabs, Hertfordshire, UK), 1 μl CoralLoad concentrate, 0.15 μl of CQ11F, 0.15 μl of molCQ11R and 0.1 μl of pipCQ11R (each at 10 pmol/μl). The thermal profile consisted of an initial denaturation step at 94 °C for 3 min followed by 35 cycles of 94 °C for 30 s, 55 °C for 30 s, 72 °C for 1 min, followed by a final elongation step at 72 °C for 10 min. Products were separated on a 1.5% agarose gel with expected product sizes of 180 bp for *Cx. pipiens* f. *pipiens*, 250 bp for *Cx. pipiens* f. *molestus*, and both bands present for *Cx. pipiens* f. *pipiens* × f. *molestus* hybrids.

#### *Anopheles maculipennis* (*s.l*.)

A subset of blood-fed mosquitoes morphologically identified as *Anopheles maculipennis* (*s.l*.) were identified to species level using the methods described in Brugman et al. [10]. Briefly, a 435 bp region of the internal transcribed spacer-2 gene (*ITS-2*) was amplified using the 5.8S and 28S primers of Collins and Paskewitz [47] in a real-time PCR assay. The final reaction mix (40 μl) consisted of: 2 μl of DNA template, 14 μl H_2_O, 20 μl SYBRGreen JumpStart Taq ReadyMix (Sigma-Aldrich) and 2 μl of each primer (at 10 pmol/μl). The thermal profile consisted of an initial denaturation step at 94 °C for 10 min followed by 35 cycles of 94 °C for 30 s, 53 °C for 30 s, 72 °C for 1 min, followed by a final elongation step of 72 °C for 10 min. PCR products were separated on a 1.5% agarose gel and positive samples sequenced unidirectionally using the ABI PRISM® BigDye® Terminator v3.1 Cycle Sequencing Kit (Applied Biosystems). Sequences were assigned to a particular mosquito species when the agreement was ≥ 98% to known species in GenBank.

### Collection of meteorological data

Meteorological data was collected at hourly intervals using an automatic weather station, data logger model CR800 (Campbell Scientific, Loughborough, UK), placed in a fixed location close to the MMP trap throughout the collection period. The variables collected were air temperature (°C), relative humidity (%), wind speed (m/s) and rainfall (mm).

### Analyses

To assess the effect of blood meal digestion stage (Sella stages II–VI) on the probability of successfully obtaining a result for blood meal host, a generalized linear mixed model (GLMM) with binomial distribution and logit link function was fitted to the data using the ‘*glmer*’ function in the ‘*lme4*’ package in R v.3.2.0 [48]. The model was fitted by maximum likelihood with the Laplace approximation, with the response being a binary variable according to obtaining either a successful or unsuccessful result following sequencing. Blood meal digestion *stage* was included as a fixed factor with five levels (each stage of digestion, Sella stages II–VI). The results for all mosquito species were combined, and thus *mosquito* was included as a random factor in the model, in a final equation *success* ~ *stage* + *mosquito*.

The effect of resting box location and meteorological variables on resting box collections of all mosquitoes, and blood-fed specimens alone, was assessed by fitting GLMMs with a negative binomial distribution to the data using the ‘*glmmADM*B’ package in R v.3.2.0 [48]. The models were fitted by maximum likelihood with the Laplace approximation and included two fixed effects (resting box *location* (A-D) and *rainfall* (mm), the latter as a binary variable of presence/absence), one random variable (*date*) and three continuous covariates (*temperature*, *relative humidity* and *wind speed*; these are hourly averages calculated from maximum values recorded each minute by the weather station), in a final equation *catch* ~ *location* + *rainfall* + *temperature* + *relative humidity* + *wind speed*. Meteorological variables were averaged over the 12 h preceding the morning collection period (20:00–08:00), the period during which feeding and flight activity leading to entry into the boxes would be expected. Multiple comparisons to assess the effect of individual factors on the response variable were made using the ‘*glht*’ function in the ‘*multcomp*’ package in R.

## Results

### Field collections

**Fig. 2:**
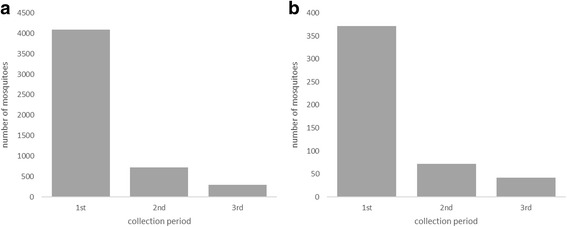
Mosquitoes collected at each collection period. The total number of (**a**) all mosquitoes and (**b**) blood-fed females (Sella stages II-VI) collected in the morning (1^st^), noon (2^nd^) and afternoon (3^rd^) collection periods

A total of 20,666 mosquitoes of eleven species were collected in the study (Table 1). Collections from the barn yielded the greatest number of mosquitoes (*n* = 13,670, 66%), followed by the resting boxes (*n* = 5,107, 25%), with blood-feds collected in similar proportions in each (10.2 and 9.5%, respectively) (see Additional file 2: Table S2 for full results broken down into collection site). The morning collection visits to the resting boxes yielded the greatest number of mosquitoes of all physiological states (*n* = 4,089, 80%) and of blood-feds specifically (*n* = 371, 75%), although mosquitoes were still collected in the noon and afternoon periods (Fig. 2).

**Table 1:** Mosquito species collected in the study. The total number of mosquitoes collected in the study by all trapping methods

Mosquito species	Total	Blood-fed (%)
*Anopheles claviger*	3	1 (33.3)
*Anopheles maculipennis* (*s.l*.)	15,653	1,671 (10.7)
*Coquillettidia richiardii*	302	10 (3.3)
*Culex modestus*	345	5 (1.4)
*Culex pipiens* (*s.l*.)*/torrentium*	1726	110 (6.4)
*Culex* spp*.*	1	0 (0)
*Culiseta annulata*	2,447	346 (14.1)
*Culiseta morsitans*	3	3 (0)
*Culiseta* spp.	25	7 (28.0)
*Ochlerotatus caspius/dorsalis*	10	0 (0)
*Ochlerotatus detritus*	18	6 (33.3)
*Ochlerotatus flavescens*	130	0 (0)
Damaged, no identification	3	0 (0)
Total	20,666	2,159 (10.4)

**Fig. 3:**
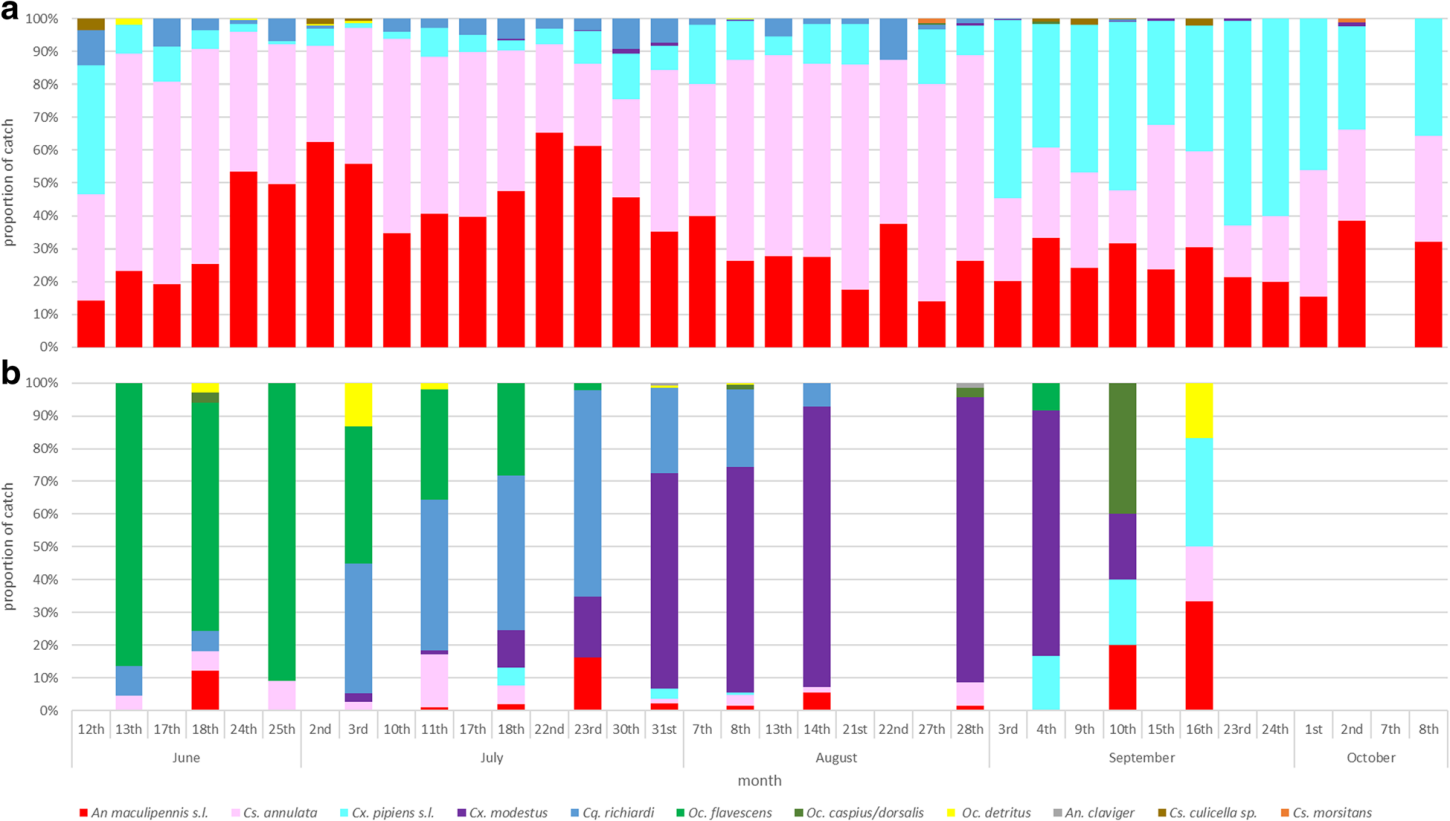
Relative abundance of mosquitoes collected June-October. Relative abundance of all mosquito species collected in (**a**) resting boxes and (**b**) in the Mosquito Magnet Pro (MMP). MMP collections were run overnight between two consecutive days of resting box collections

*Anopheles maculipennis* (*s.l*.) represented the largest proportion of the catch overall (*n* = 15,653), followed by *Culiseta annulata* (*n* = 2,447) and *Cx. pipiens* (*s.l*.)*/torrentium* (*n* = 1,726). These three dominated the collections from the resting boxes throughout the collection period as compared to the Mosquito Magnet Pro trap wherein a sequential dominance of *Ochlerotatus flavescens* (June - mid-July), *Coquillettida richiardii* (early July - early August) and then *Culex modestus* (mid-July - mid-September) was observed (Fig. 3).

### Impact of meteorological variables and resting box location on collections

Resting box locations yielded 3,488 (A), 248 (B), 1,205 (C) and 166 (D) mosquitoes (males and females, all physiological states) overall. At each location blood-fed mosquitoes comprised a respective 10% (*n* = 360), 9% (*n* = 22), 7% (*n* = 87) and 5% (*n* = 9) of the totals. Multiple comparisons indicated that resting box location significantly influenced the number of mosquitoes collected (total (TM) and blood-fed (BF) alone) when comparing locations B - A (TM: *Z* = -20.91, *P* < 0.0001; BF: *Z* = -10.39, *P* < 0.0001), C - A (TM: *Z* = -10.65, *P* < 0.0001; BF: *Z* = -8.72, *P* < 0.0001), D - A (TM: *Z* = -21.61, *P* < 0.0001; BF: *Z* = -9.70, *P* < 0.0001), C - B (TM: *Z* = 9.78, *P* < 0.0001; BF: *Z* = 4.94, *P* < 0.0001) and D - C (TM: *Z* = -9.34, *P* < 0.0001; BF: *Z* = -4.14, *P* < 0.0001). Locations B and D did not differ significantly for either total mosquitoes or blood-fed mosquitoes (Additional file 3: Table S3). All recorded meteorological variables influenced the total mosquitoes collected: wind speed (*Z* = -4.13, *P* < 0.0001), temperature (*Z* = 3.73, *P* = 0.00019), relative humidity (*Z* = 2.32, *P =* 0.021) and the presence of rainfall (*Z* = -2.14, *P* = 0.032). Only wind speed and temperature influenced the collections of blood-fed mosquitoes: over the night preceding the morning collection period, an increase of 1 m/s average wind speed was predicted to lead to an estimated 51% fewer blood-feds in the resting boxes (*Z* = -3.13, *P* = 0.0018) whilst a 1 °C increase in temperature would lead to an expected 127% more mosquitoes collected (*Z* = 4.08, *P* < 0.0001) (Additional file 3: Table S3).

### Blood meal analysis results

Blood-fed specimens (Sella stages II-VI, *n* = 2,159) constituted 10.4% of the total collected mosquitoes, with unfed females (*n* = 8,493, 41%) and males (*n* = 8,285, 40%) comprising the majority (Additional file 4: Table S4). A total of 1,341 blood-feds were selected for analysis, of which 964 (72%) successfully produced a result for blood meal origin, a rate of 77% at the PCR amplification stage (range 46–100%) and 93% at the sequencing stage (range 52–100%) (Additional file 5: Table S5). When compared to freshly blood-fed specimens (Sella stage II), only mosquitoes (all species) of stage V (odds ratio, OR = 0.19, *Z* = -3.14, *P* = 0.0017) and VI (OR = 0.04, *Z* = -6.76, *P* < 0.0001) led to a significantly reduced probability of obtaining a successful blood meal identification (Additional file 6: Table S6).

Nineteen host species comprising five mammals (Table 2) and fourteen birds (Table 3) were identified from mosquito blood meals. Two of the bird species, the barn swallow (*Hirundo rustica* L.), fed on by *Cx. pipiens* f. *pipiens* and *Cx. modestus*, and yellow wagtail (*Motacilla flava* L.), fed on by *Cx. pipiens* f. *pipiens* are summer migrants to the UK; the remaining 12 birds are resident species. *Anopheles maculipennis* (*s.l*.) and *Cx. pipiens* f. *pipiens* were found to have fed on dark-breasted barn owl (*Tyto alba* Scopoli subsp. *guttata*), a variant not previously known to occur in Kent. *Culex pipiens* f. *pipiens* fed only on birds, with the barn swallow and wood pigeon (*Columba palumbus* L.) being the most dominant hosts among the 13 identified bird species. Four specimens of *Cx. modestus* were identified as having fed on two species of bird, the mute swan (*Cygnus olor* Gmelin) and barn swallow.

**Table 2:** Mosquito species with mammalian blood meals

Mosquito species	Mammals					Total
	Brown rat	Cow	European hare	European rabbit	Sheep	
*An. claviger*	0	1	0	0	0	1
*An. maculipennis* (*s.l*.)^a^	2	344	2	228	6	582
*Cq. richiardii*	0	3	0	0	0	3
*Cs. annulata*	0	187	0	4	5	196
*Oc. detritus*	0	2	0	0	0	2
*Culiseta* spp.^a^	0	1	0	0	0	1
Total	2	538	2	232	11	785

Mammalian blood meals identified from the mosquito species in this study

^a^Indicates species that also fed on birds (see Table 3)

**Table 3:** Mosquito species with avian blood meals

Mosquito species	Bird species														Total
	Blackbird	Barn swallow	Chicken	Dark-breasted barn owl	Eurasian skylark	European starling	Grey heron	House sparrow	Long-eared owl	Meadow pipit	Mute swan	Stock dove	Wood pigeon	Yellow wagtail	
*An. maculipennis* (*s.l*.)^a^	0	0	60	1	0	0	0	0	0	0	0	10	0	0	71
*Cx. modestus*	0	3	0	0	0	0	0	0	0	0	1	0	0	0	4
*Cx. pipiens* f. *pipiens*	19	92	1	3	2	1	3	12	1	2	0	1	42	1	97
*Cs. morsitans*	2	0	0	0	0	0	0	1	0	0	0	0	0	0	3
*Culiseta* spp.^a^	1	0	0	0	0	0	0	1	0	0	0	0	1	0	3
Unknown (damaged)	0	0	0	0	0	0	0	0	0	0	0	0	1	0	1
Total	22	95	61	4	2	1	3	14	1	2	1	11	44	1	262

Avian blood meals identified from the mosquito species in this study

^a^indicates species that also fed on mammals (see Table 3)

A total of 149 specimens of *An. maculipennis* (*s.l*.) were identified by molecular methods to species level, resulting in 83 *Anopheles atroparvus* specimens and 66 specimens returning identical results (100% identity in BLAST searches) for *Anopheles daciae* and *Anopheles messeae* (Table 4). *Anopheles daciae/messeae* and *An. atroparvus* both fed on mammals and birds, the latter group comprising chickens (*Gallus gallus* L.) and stock doves (*Columba oenas* L.) for *An. atroparvus*, and only chickens for *An. daciae/messeae*.

**Table 4:** Vertebrate blood meals of *Anopheles atroparvus* and *Anopheles daciae/messeae*

Mosquito species	Barn owl	Brown rat	Chicken	Cow	Hare	Rabbit	Sheep	Stock dove	Total
*An. atroparvus*	0	2	16	30	1	22	2	10	83
*An. daciae/messeae*	1	0	23	37	1	0	4	0	66
Total	1	2	39	67	2	22	6	10	149

Vertebrate blood meal hosts of a subset of *Anopheles maculipennis* (*s.l*.) identified by molecular methods as *An. atroparvus* or *An. daciae/messeae*; the latter species grouping is presented as such as specimens produced a 100% identity to both species in BLAST results

Small-scale variations in trapping location appeared to influence the blood meal hosts identified, illustrated by the results for *An. maculipennis* (*s.l*.). The majority of specimens (54/55, 98%) collected from the chicken coop had fed on chickens, whilst chicken blood meals represented a low proportion in the toilets (3/112, 3%) and in resting boxes at location A (2/51, 4%) and C (1/11, 9%) although these were less than 50 m away. In contrast, cattle and rabbits were widely distributed across the site, reflected in the appearance of mosquitoes that had fed on these hosts in all except the chicken coop and resting box location D, respectively.

All raw data have been included as additional files accompanying this manuscript (Additional file 7: Table S7).

## Discussion

This study investigated the vertebrate blood meal hosts of mosquitoes from a Thames estuary wetland region in southeast England. Nine blood-fed mosquito species were collected, from which nineteen blood meal host species were identified, comprising five mammals and fourteen birds. Both resident and migratory bird species were blood meal hosts for two important potential arbovirus vectors, *Cx. pipiens* f. *pipiens* and *Cx. modestus*. The feeding behaviour of these potential vectors could, therefore, facilitate the enzootic transmission of an introduced, exotic arbovirus in the local area.

Migratory birds have not previously been identified as blood meal hosts in the UK. However, their possible role in the long-distance translocation of arboviruses such as WNV from endemic regions has been extensively discussed [49–51], and they are targeted as part of arbovirus surveillance programmes across Europe [28, 52]. Barn swallows were the most utilised avian host overall in this study (n = 95) (Table 3). Members of this species are long-distance migrants which over-winter in southern Africa and have been identified as having been exposed to WNV in Germany [52]. Indeed, several birds identified as blood meal hosts in this study are highly susceptible to infection with arboviruses. These include the two owl species identified (Table 3); owls have been associated with high levels of mortality in European outbreaks of USUTV [53–55]. The dark-breasted barn owl, *Tyto alba gutatta*, is considered an infrequent visitor to the UK from its range in mainland Europe [56]. Although several individuals have previously been recorded in East Anglia [56], records for this variant do not exist for the study site. This provides an interesting insight into using blood meal analysis to monitor specific species within a community, as demonstrated for the Iberian lynx in Spain [57].

The PCR-sequencing approach used in this study was successful in identifying host origin in 72% of blood-fed mosquitoes, with a decrease in success rates for all three of the most numerous species, *An. maculipennis* (*s.l*.), *Cs. annulata* and *Cx. pipiens* (*s.l*.), as Sella stage increased from stage II through to VI (Additional file 5: Table S5). This result is consistent with a previous study on field-collected specimens elsewhere [34] using a different PCR assay [58]. The major limitation with the methodology that was used in this study is that no mixed feeds could be detected, as the most abundant blood meal source will dominate the PCR reaction. An additional consideration is the storage method for specimens; storage of samples at −20 °C may lead to poor blood meal amplification results [59], a factor which was not considered in the present study.

This study found four mosquito species that fed exclusively on mammals, three only on birds, and two that fed on both host groups. The structure of hosts within an ecological community can influence pathogen transmission dynamics [60], and understanding which hosts are utilised by mosquitoes within particular ecological settings is, therefore, important. *Culiseta annulata* fed predominantly on cattle in this study. This corresponds with other studies showing predominant mammal-feeding [8, 61] although, in the previous UK study, non-specific avian feeding was also detected. This suggests that *Cs. annulata* could make an effective vector for mosquito-borne viruses that infect livestock, such as Rift Valley fever virus (RVFV) [62]. Although this species has been implicated as a potential vector [3], there is no experimental data to confirm this assertion, and this could be a focus for future study. By contrast, anopheline species have been considered poor vectors for RVFV [63], and so despite feeding on cattle in this study, they may not contribute to disease transmission.

A complex interplay of mosquito-, host- and environmentally-associated factors influence mosquito feeding patterns [64]. Among these, host availability is a major driver of host selection by mosquitoes [18, 65]. The availability of hosts to mosquito feeding is affected by their relative abundance in the community in addition to other factors such as host defensive behaviour [15, 66]. In this study, the presence of a host at or close to a particular collection site may have increased its availability to mosquitoes nearby and therefore have led to increased feeding rates. For example, 98% of specimens of *An. maculipennis* (*s.l*.) collected in the chicken coop had fed upon chickens, whilst the proportion of chicken blood meals in specimens collected further away from the chicken coops was much lower (< 10%). All the birds fed upon by *An. maculipennis* (*s.l*.) were available ‘indoors’ either resting and/or nesting in the barn (stock doves, barn owls) or inside the chicken coops (chickens), indicating that these hosts may have been selected due to proximity to indoor resting sites. Specimens of *An. maculipennis* (*s.l*.) delineated to species level showed *An. daciae/messeae* fed on both birds and mammals, in agreement with a previous UK study [9]. However, *An. atroparvus* has not previously been identified as feeding on birds in the UK, although a recent study in Spain detected a low frequency of feeding on chickens (2/115, 1.7%), but no other avian species [34].

The Thames estuary region had a long association with human-biting mosquitoes and associated *P. vivax* transmission [35–37]. Therefore, the absence of human blood meals in the study, especially from malaria vectors such as *An. atroparvus*, is perhaps surprising. However, this finding may also reflect a low human host availability, as the Elmley site closes to visitors at sunset, the peak period of mosquito activity on site [7, 35]. Furthermore, residents on site report that they sometimes sleep under bed nets and indeed bed nets are utilised as personal protection from mosquito biting more widely across the local area [36]. A further reason for the absence of human blood meals is the bias towards endophilic species by the primary collection methods used in this study. This is evident when comparing the species profiles of Mosquito Magnet collections, which targeted the host-seeking component of the population, to those of the resting boxes (Fig. 3). Future studies utilising multiple trap types to target several subsets of the adult mosquito population, similar to trapping conducted recently in Germany [18], may yield a wider range of blood-fed species including those containing human blood meals.

*Culex pipiens* f. *pipiens* was found to have only taken blood meals from birds in this study. Predominant avian feeding by this species corresponds with the findings of other studies that have separated the ecoforms of *Cx. pipiens* (*s.l*.) elsewhere in Europe [67], although mammalian meals were also reported there. Here, barn swallows, wood pigeon and blackbirds were found to be the most utilised hosts for *Cx. pipiens* f. *pipiens*. A recent study in Italy found blackbirds were among the most utilised hosts relative to their abundance and blackbird-derived odours were preferred over the odours of other species in laboratory assays with *Cx. pipiens* (*s.l*.) [68]. However, in the absence of detailed host surveys in this study, conclusions regarding the host preference of this species cannot be drawn.

Despite the aforementioned species biases of resting collections, this study has demonstrated that resting boxes can successfully be used in a UK field situation to collect large numbers of blood-fed specimens, despite limited success elsewhere [69]. This success coupled with the simplicity of construction and portability of these traps will hopefully encourage the use of this design in future studies. The reason for the high levels of success with the boxes is not clear but is likely due to a combination of the intensity of collections, three times a day on each of the 36 collection visits, as well as their use in an area where three endophilic species are found at high population density. It is interesting to note that daytime recruitment of mosquitoes into the boxes occurred even after complete clearance of mosquitoes in earlier collection periods (Fig. 2). Whether this is as a result of daytime feeding activity or disturbance of already blood-fed individuals from nearby outdoor resting sites (or both) is uncertain, but this does indicate that multiple daily collection visits to a resting box can be productive. Furthermore, the greatest collections were from the boxes placed in the central woodland strip (location A), one of the few areas providing structured vegetation in the immediate area (Fig. 1). Given the importance of meteorological variables in influencing resting box collections (Additional file 3: Table S3), the shelter provided by vegetation in this area may have increased the density of mosquitoes in the immediate area and correspondingly entry into the boxes.

## Conclusions

Mosquito species in this study were demonstrated to feed on both resident and migratory bird species in a coastal grazing marsh on the southeast coast of England, and thus could provide a transmission pathway that could lead to the establishment and spread of enzootic arboviruses in the area. The feeding patterns observed indicate that *Cx. pipiens* f. *pipiens* and *Cx. modestus* could play a key role in establishing bird-associated viruses such as West Nile virus and Sindbis virus. However, no evidence for these species feeding on mammals, including humans, was identified in this study and so they may be unlikely to cause spillover infection. Alternatively, the opportunistic feeding behaviour observed from *An. maculipennis* (*s.l*.) suggests that this species group deserves greater attention as a potential bridge vector for pathogens between avian and mammalian hosts. Large-scale collections of blood-fed mosquitoes were proved successful by intensively targeting resting mosquito populations. Future work should aim to develop more effective techniques for the collection of species groups which rest outdoors; the resulting information on blood-feeding patterns of these groups will, together with emerging data on vector competence, provide a more complete picture of the risk of pathogen transmission in the event of an incursion.

## Additional files

Additional file 1: Table S1. Details of all primer sets used in this study. (PDF 98 kb)

Additional file 2: Table S2. Full breakdown of mosquito field collection results over the 36 collection visits by trap type and location. (PDF 41 kb)

Additional file 3: Table S3. (**a**) Regression coefficients, plus Wald 95% confidence intervals, standard errors and Z values, for the negative binomial GLMM for all mosquitoes (all species, all physiological states). Predicted % difference is the (exponent × 100) of the value in the estimate column and gives the estimated change in blood-fed numbers collected depending on the box location compared to resting box location A as a baseline, or for a one-unit increase in meteorological variables. ***P ≤ 0.001, **P ≤ 0.01, *P ≤ 0.05. (**b**) Multiple Tukey’s comparisons between the numbers of mosquitoes (all species, all physiological states) collected in each resting box location for all species. Predicted % difference is the (exponent × 100) of the value in the estimate column and gives the estimated change of the catch between two resting boxes. ***P ≤ 0.001. (**c**) Regression coefficients, plus Wald 95% confidence intervals, standard errors and Z values, for the final negative binomial GLMM, for all blood-fed mosquito species. Predicted % difference is the (exponent × 100) of the value in the estimate column and gives the estimated change in blood-fed numbers collected depending on the box location compared to resting box location A as a baseline, or for a one-unit increase in meteorological variables. ***P ≤ 0.001, **P ≤ 0.01. (**d**) Description: Multiple Tukey’s comparisons between the numbers of blood-feds collected in each resting box location for all species. Predicted % difference is the (exponent × 100) of the value in the estimate column and gives the estimated change of the catch between two resting boxes. ***P ≤ 0.001. (PDF 136 kb)

Additional file 4: Table S4. Mosquito species organised according to Sella stage of digestion. Mosquitoes comprise all specimens collected in the study (all trap locations and by all collection methods combined across 36 visits to Elmley in 2014). (PDF 24 kb)

Additional file 5: Table S5. Success rates for blood meal identification for blood-fed specimens of three mosquito species at increasing stages of digestion (Sella stages II – VI) at the PCR and sequencing steps of the analysis workflow. ‘All species’ refers to the three species included in the table plus the remaining species included in the overall blood meal analysis. (PDF 123 kb)

Additional file 6: Table S6. Binomial GLMM regression coefficients, with 95% Wald confidence intervals, standard error and Z values, for the likelihood of successfully obtaining a vertebrate host blood meal identification at increasing Sella stages of digestion. The odds ratios are the exponent of the values in the ‘estimate’ column and indicate the odds of successful identification in comparison to a mosquito with a blood meal at Sella stage II. ***P ≤ 0.001, **P ≤ 0.01. (PDF 250 kb)

Additional file 7: Table S7. Raw data collected in the study. (XLSX 934 kb)
