# Supplementary material for: Blood-feeding patterns of native mosquitoes and insights into their potential role as pathogen vectors in the Thames estuary region of the United Kingdom
Source: Parasit Vectors. 2017 Mar 27;10:163. doi: 10.1186/s13071-017-2098-4 (PMC5369192; doi:10.1186/s13071-017-2098-4)
Supplement: Supplementary file 1 — Details of all primer sets used in this study. (PDF 98 kb) [file 13071_2017_2098_MOESM1_ESM.pdf]

Additional file 1: Table S1. Details of all primer sets used in this study

Purpose	Primer name	Primer sequence (5' – 3')	Reference(s)
Identification of vertebrate blood meal	VF1_t1	TGTAAAACGACGGCCAGTTCTCAACCAACCACAAAGACATTGG	42
	VF1d_t1	TGTAAAACGACGGCCAGTTCTCAACCAACCACAARGAYATYGG	
	VF1i_t1	TGTAAAACGACGGCCAGTTCTCAACCAACCAIAAIGAIATIGG	
	VR1d_t1	CAGGAAACAGCTATGACTAGACTTCTGGGTGGCCRAARAAYCA	
	VR1_t1	CAGGAAACAGCTATGACTAGACTTCTGGGTGGCCAAAGAATCA	
	VR1i_t1	CAGGAAACAGCTATGACTAGACTTCTGGGTGICCIAAIAAICA	
Separation of *Culex pipiens* s.l. and *Culex torrentium*	ACEtorr	TGCCTGTGCTACCAGTGATGTT	44
	ACEpip	GGAAACAACGACGTATGTAG	
Separation of *Culex pipiens* form *pipiens* and *Culex pipiens* form *molestus*	CQ11F	GATCCTAGCAAGCGAGAAC	45, 46
	molCQ11	CCCTCCAGTAAGGTATCAAC	
	pipCQ11R	CATGTTGAGCTTCGGTGAA	
Species delineation of *Anopheles maculipennis* s.l.	5.8S	TGTGAACTGCAGGACACAT	47
	28S	ATGCTTAAATTTAGGGGGTA	
