# Supplementary material for: Blood-feeding patterns of native mosquitoes and insights into their potential role as pathogen vectors in the Thames estuary region of the United Kingdom
Source: Parasit Vectors. 2017 Mar 27;10:163. doi: 10.1186/s13071-017-2098-4 (PMC5369192; doi:10.1186/s13071-017-2098-4)
Supplement: Supplementary file 2 — Full breakdown of mosquito field collection results over the 36 collection visits by trap type and location. (PDF 41 kb) [file 13071_2017_2098_MOESM2_ESM.pdf]

Additional file 2: Table S2. Full breakdown of mosquito field collection results over the 36 collection visits by trap type and location.

**Mosquito species**	***Resting boxes***		***Toilets***		***Barn***		***Chicken coops***		***Roof structure***		***Red feeder***		***MMP***		***Totals***	
	total	*BF (%)*	*total*	*BF (%)*	*Total*	*BF (%)*	*total*	*BF (%)*	*total*	*BF (%)*	*total*	*BF (%)*	*total*	*BF (%)*	***total***	***BF (%)***
Anopheles claviger	0	-	0	-	1	1 (100)	0	-	0	-	0	-	2	0 (0)	**3**	**1 (33.3)**
Anopheles maculipennis s.l.	1809	95 (5.3)	776	166 (21.4)	12832	1329 (10.4)	131	73 (55.7)	21	1 (4.8)	59	7 (11.9)	25	0 (0)	**15653**	**1671 (10.7)**
Coquillettidia richiardii	106	9 (8.5)	2	0 (0)	1	0 (0)	0	-	0	-	0	-	193	1 (0.5)	**302**	**10 (3.3)**
Culex modestus	14	2 (14.3)	6	3 (50)	1	0 (0)	0	-	0	-	0	-	324	0 (0)	**345**	**5 (1.4)**
Culex pipiens s.l./torrentium	1038	85 (8.2)	72	13 (18.1)	567	11 (1.9)	0	-	35	1 (2.9)	1	0 (0)	13	0 (0)	**1726**	**110 (6.4)**
Culex spp.	1	0 (0)	0	-	0	-	0	-	0	-	0	-	0	-	**1**	**0 (0)**
Culiseta annulata	2104	279 (13.3)	33	7 (21.2)	267	57 (21.3)	0	-	2	2 (100)	1	0 (0)	40	1 (2.5)	**2447**	**346 (14.1)**
Culiseta morsitans	3	3 (100)	0	-	0	-	0	-	0	-	0	-	0	-	**3**	**3 (0)**
Culiseta spp.	25	7 (28.0)	0	-	0	-	0	-	0	-	0	-	0	-	**25**	**7 (28.0)**
Ochlerotatus caspius/dorsalis	2	0 (0)	1	0 (0)	0	-	0	-	0	-	0	-	7	0 (0)	**10**	**0 (0)**
Ochlerotatus detritus	5	5 (100)	0	-	1	1 (100)	0	-	1	0 (0)	0	-	11	0 (0)	**18**	**6 (33.3)**
Ochlerotatus flavescens	0	-	0	-	0	-	0	-	0	-	0	-	130	0 (0)	**130**	**0 (0)**
Damaged	-	-	-	-	-	-	-	-	-	-	-	-	-	-	**3**	**0 (0)**
Totals	**5107**	**485 (9.5)**	**890**	**189 (21.2)**	**13670**	**1399 (10.2)**	**131**	**73 (55.7)**	**59**	**4 (6.8)**	**61**	**7 (11.5)**	**745**	**2 (0.3)**	**20666**	**2159 (10.4)**
