# Supplementary material for: Blood-feeding patterns of native mosquitoes and insights into their potential role as pathogen vectors in the Thames estuary region of the United Kingdom
Source: Parasit Vectors. 2017 Mar 27;10:163. doi: 10.1186/s13071-017-2098-4 (PMC5369192; doi:10.1186/s13071-017-2098-4)
Supplement: Supplementary file 3 — (a) Regression coefficients, plus Wald 95% confidence intervals, standard errors and Z values, for the negative binomial GLMM for all mosquitoes (all species, all physiological states). Predicted % difference is the (exponent × 100) of the value in the estimate column and gives the estimated change in blood-fed numbers collected depending on the box location compared to resting box location A as a baseline, or for a one-unit increase in meteorological variables. ***P ≤ 0.001, **P ≤ 0.01, *P ≤ 0.05. (b) Multiple Tukey’s comparisons between the numbers of mosquitoes (all species, all physiological states) collected in each resting box location for all species. Predicted % difference is the (exponent × 100) of the value in the estimate column and gives the estimated change of the catch between two resting boxes. ***P ≤ 0.001. (c) Regression coefficients, plus Wald 95% confidence intervals, standard errors and Z values, for the final negative binomial GLMM, for all blood-fed mosquito species. Predicted % difference is the (exponent × 100) of the value in the estimate column and gives the estimated change in blood-fed numbers collected depending on the box location compared to resting box location A as a baseline, or for a one-unit increase in meteorological variables. ***P ≤ 0.001, **P ≤ 0.01. (d) Description: Multiple Tukey’s comparisons between the numbers of blood-feds collected in each resting box location for all species. Predicted % difference is the (exponent × 100) of the value in the estimate column and gives the estimated change of the catch between two resting boxes. ***P ≤ 0.001. (PDF 136 kb) [file 13071_2017_2098_MOESM3_ESM.pdf]

Additional file 3: Table S3. (**a**) Regression coefficients, plus Wald 95% confidence intervals, standard errors and z values, for the negative binomial GLMM for all mosquitoes (all species, all physiological states). Predicted % difference is the (exponent × 100) of the value in the estimate column and gives the estimated change in blood-fed numbers collected depending on the box location compared to resting box location A as a baseline, or for a one-unit increase in meteorological variables. ***$P \leq 0.001$, **$P \leq 0.01$, *$P \leq 0.05$. (**b**) Multiple Tukey's comparisons between the numbers of mosquitoes (all species, all physiological states) collected in each resting box location for all species. Predicted % difference is the (exponent x 100) of the value in the estimate column and gives the estimated change of the catch between two resting boxes. ***$P \leq 0.001$. (**c**) Regression coefficients, plus Wald 95% confidence intervals, standard errors and z values, for the final negative binomial GLMM, for all blood-fed mosquito species. Predicted % difference is the (exponent x 100) of the value in the estimate column and gives the estimated change in blood-fed numbers collected depending on the box location compared to resting box location A as a baseline, or for a one-unit increase in meteorological variables. ***$P \leq 0.001$, **$P \leq 0.01$. (**d**) Description: Multiple Tukey's comparisons between the numbers of blood-feds collected in each resting box location for all species. Predicted % difference is the (exponent x 100) of the value in the estimate column and gives the estimated change of the catch between two resting boxes. ***$P \leq 0.001$.

(a)

**Coefficients**	**Estimate (95% CI)**	**Predicted % difference**	**Std. Error**	**Z value**
(Intercept)	-4.929 (-9.98; 0.03)	-	2.5290	-1.95
location B	-2.572 (-2.81; -2.33) ***	7.64	0.1230	-20.91
location C	-1.074 (-1.27; -0.88) ***	34.16	0.1008	-10.65
location D	-2.819 (-3.07; -2.56) ***	5.97	0.1305	-21.61
rainfall	-0.699 (-1.34; -0.06) *	49.71	0.3266	-2.14
wind speed	-0.767 (-1.13; -0.40) ***	46.44	0.1858	-4.13
temperature	0.200 (0.09; 0.31) ***	122.14	0.0537	3.73
relative humidity	0.061 (0.01; 0.11) *	106.29	0.0262	2.32

(b)

Linear hypotheses: Resting box comparison	Estimate	Predicted % difference	Std. Error	Z value
B - A == 0	-2.572 ***	-7.64	0.1230	-20.909
C - A == 0	-1.074 ***	-34.16	0.1008	-10.653
D - A == 0	-2.819 ***	-5.97	0.1305	-21.606
C - B == 0	1.498 ***	447.27	0.1532	9.779
D - B == 0	-0.247	-78.66	0.1922	-1.286
D - C == 0	-1.745 ***	-17.46	0.1868	-9.344

(c)

Coefficients	Estimate (95% CI)	Predicted % difference	Std. Error	Z value
(Intercept)	-3.149 (-8.31; 2.01)	-	2.63170	-1.20
location B	-2.849 (-3.39; -2.31) ***	-5.79	0.27429	-10.39
location C	-1.431 (-1.75; -1.11) ***	-23.92	0.16409	-8.72
location D	-3.415 (-4.11; -2.73) ***	-3.29	0.35207	-9.70
rainfall	-0.379 (-1.07; 0.31)	-68.48	0.35050	-1.08
wind speed	-0.679 (-1.10; -0.25) **	-50.73	0.21707	-3.13
temperature	0.243 (0.13; 0.36) ***	127.47	0.05945	4.08
relative humidity	0.006 (-0.05; 0.06)	100.61	0.02752	0.22

(d)

Linear hypotheses: Resting box comparison	Estimate	Predicted % difference	Std. Error	Z value
B - A == 0	-2.849 ***	-5.79	0.2743	-10.386
C - A == 0	-1.431 ***	-23.92	0.1641	-8.719
D - A == 0	-3.415 ***	-3.29	0.3521	-9.700
C - B == 0	1.418 ***	412.93	0.2869	4.943
D - B == 0	-0.566	-56.76	0.5174	-1.095
D - C == 0	-1.985 ***	-13.74	0.4799	-4.135
