# Supplementary material for: Blood-feeding patterns of native mosquitoes and insights into their potential role as pathogen vectors in the Thames estuary region of the United Kingdom
Source: Parasit Vectors. 2017 Mar 27;10:163. doi: 10.1186/s13071-017-2098-4 (PMC5369192; doi:10.1186/s13071-017-2098-4)
Supplement: Supplementary file 4 — Mosquito species organised according to Sella stage of digestion. Mosquitoes comprise all specimens collected in the study (all trap locations and by all collection methods combined across 36 visits to Elmley in 2014). (PDF 24 kb) [file 13071_2017_2098_MOESM4_ESM.pdf]

Additional file 4: Table S4. Mosquito species organised according to Sella stage of digestion. Mosquitoes comprise all specimens collected in the study (all trap locations and by all collection methods combined across 36 visits to Elmley in 2014).

Mosquito species	Males (%)	Sella stage of blood meal digestion							Totals
		I (%)	II (%)	III (%)	IV (%)	V (%)	VI (%)	VII (%)	
Anopheles claviger	0 (0)	2 (66.7)	1 (33.3)	0 (0)	0 (0)	0 (0)	0 (0)	0 (0)	**3**
Anopheles maculipennis s.l.	6294 (40.2)	6655 (42.5)	171 (1.1)	420 (2.7)	408 (2.6)	340 (2.2)	332 (2.1)	1033 (6.6)	**15 653**
Coquillettidia richiardii	45 (14.9)	216 (71.5)	1 (0.3)	0 (0)	3 (1.0)	3 (1.0)	3 (1.0)	31 (10.3)	**302**
Culex modestus	1 (0.3)	336 (97.4)	1 (0.3)	1 (0.3)	2 (0.6)	1 (0.3)	0 (0)	3 (0.9)	**345**
Culex pipiens s.l./torrentium	673 (39.0)	524 (30.4)	42 (2.4)	40 (2.3)	15 (0.9)	3 (0.2)	10 (0.6)	419 (24.3)	**1726**
Culex spp.	0 (0)	1 (100)	0 (0)	0 (0)	0 (0)	0 (0)	0 (0)	0 (0)	**1**
Culiseta annulata	1267 (51.8)	601 (24.6)	32 (1.3)	113 (4.6)	80 (3.3)	68 (2.8)	53 (2.2)	233 (9.5)	**2447**
Culiseta morsitans	0 (0)	0 (0)	2 (66.7)	1 (33.3)	0 (0)	0 (0)	0 (0)	0 (0)	**3**
Culiseta spp.	4 (16.0)	5 (20.0)	0 (0)	3 (12.0)	2 (8.0)	0 (0)	2 (8.0)	9 (36.0)	**25**
Ochlerotatus caspius/dorsalis	1 (10.0)	9 (90.0)	0 (0)	0 (0)	0 (0)	0 (0)	0 (0)	0 (0)	**10**
Ochlerotatus detritus	0 (0)	11 (61.1)	1 (5.6)	3 (16.7)	2 (11.1)	0 (0)	0 (0)	1 (5.6)	**18**
Ochlerotatus flavescens	0 (0)	130 (100)	0 (0)	0 (0)	0 (0)	0 (0)	0 (0)	0 (0)	**130**
Damaged (not identifiable)	0 (0)	3 (100)	0 (0)	0 (0)	0 (0)	0 (0)	0 (0)	0 (0)	**3**
**Totals**	**8285 (40.1)**	**8493 (41.1)**	**251 (1.2)**	**581 (2.8)**	**512 (2.5)**	**415 (2.0)**	**400 (1.9)**	**1729 (8.4)**	**20 666**
