# Supplementary material for: Blood-feeding patterns of native mosquitoes and insights into their potential role as pathogen vectors in the Thames estuary region of the United Kingdom
Source: Parasit Vectors. 2017 Mar 27;10:163. doi: 10.1186/s13071-017-2098-4 (PMC5369192; doi:10.1186/s13071-017-2098-4)
Supplement: Supplementary file 5 — Success rates for blood meal identification for blood-fed specimens of three mosquito species at increasing stages of digestion (Sella stages II – VI) at the PCR and sequencing steps of the analysis workflow. ‘All species’ refers to the three species included in the table plus the remaining species included in the overall blood meal analysis. (PDF 123 kb) [file 13071_2017_2098_MOESM5_ESM.pdf]

Additional file 5: Table S5. Success rates for blood meal identification for blood-fed specimens of three mosquito species at increasing stages of digestion (Sella stages II – VI) at the PCR and sequencing steps of the analysis workflow. 'All species' refers to the three species included in the table plus the remaining species included in the overall blood meal analysis.

**Processing stages**	**All species**	***An. maculipennis* s.l.**					***Culiseta annulata***					***Culex pipiens* s.l.**				
	all stages	*II*	*III*	*IV*	*V*	*VI*	*II*	*III*	*IV*	*V*	*VI*	*II*	*III*	*IV*	*V*	*VI*
Total mosquitoes tested	1341	170	415	111	81	81	31	112	81	66	56	42	35	16	2	9
PCR positive	1034	153	348	92	56	62	25	68	51	37	24	41	33	15	2	6
**PCR success rate**	**0.77**	**0.90**	**0.84**	**0.83**	**0.69**	**0.77**	**0.81**	**0.61**	**0.63**	**0.56**	**0.43**	**0.98**	**0.94**	**0.94**	**1.00**	**0.67**
Successful blood meal ID	964	152	337	88	47	30	22	67	51	35	21	40	33	15	10	4
**Sequencing success rate**	**0.93**	**0.99**	**0.97**	**0.96**	**0.84**	**0.48**	**0.88**	**0.99**	**1.00**	**0.95**	**0.88**	**0.98**	**1.00**	**1.00**	**1.00**	**0.67**
**Final success rate**	**0.72**	**0.89**	**0.81**	**0.80**	**0.58**	**0.37**	**0.71**	**0.60**	**0.63**	**0.53**	**0.38**	**0.96**	**0.94**	**0.94**	**1.00**	**0.45**
**%**	**72**	**89**	**81**	**80**	**58**	**37**	**71**	**60**	**63**	**53**	**38**	**96**	**94**	**94**	**100**	**45**
