# Supplementary material for: Blood-feeding patterns of native mosquitoes and insights into their potential role as pathogen vectors in the Thames estuary region of the United Kingdom
Source: Parasit Vectors. 2017 Mar 27;10:163. doi: 10.1186/s13071-017-2098-4 (PMC5369192; doi:10.1186/s13071-017-2098-4)
Supplement: Supplementary file 6 — Binomial GLMM regression coefficients, with 95% Wald confidence intervals, standard error and Z values, for the likelihood of successfully obtaining a vertebrate host blood meal identification at increasing Sella stages of digestion. The odds ratios are the exponent of the values in the ‘estimate’ column and indicate the odds of successful identification in comparison to a mosquito with a blood meal at Sella stage II. ***P ≤ 0.001, **P ≤ 0.01. (PDF 250 kb) [file 13071_2017_2098_MOESM6_ESM.pdf]

Additional file 6: Table S6. Binomial GLMM regression coefficients, with 95% Wald confidence intervals, standard error and Z values, for the likelihood of successfully obtaining a vertebrate host blood meal identification at increasing Sella stages of digestion. The odds ratios are the exponent of the values in the 'estimate' column and indicate the odds of successful identification in comparison to a mosquito with a blood meal at Sella stage II. *** $P \leq 0.001$, ** $P \leq 0.01$.

Coefficients:	Estimate (95% CI)	Odds Ratio	Std. Error	Z value
(Intercept)	3.975 (2.94; 5.01) ***	-	0.529633	7.504
stage 3	-0.095 (-1.07; 0.87)	0.91	0.494845	-0.193
stage 4	0.002 (-1.28; 1.28)	1.00	0.654019	0.003
stage 5	-1.659 (-2.69; -0.62) **	0.19	0.528356	-3.138
stage 6	-3.184 (-4.11; -2.26) ***	0.04	0.470784	-6.761
